# Toward Cartilage-Mimicking Biomaterials: Biotribological, Biochemical and Structural Evaluation of pHEMA and PVA-Based Hydrogels

**DOI:** 10.1021/acsomega.5c10283

**Published:** 2025-12-08

**Authors:** David Nečas, Daniel Němeček, Jan Gregora, David Rebenda, Zuzana Kadlecová, Ivana Chamradová, Monika Trudičová, Pavel Čípek, Petr Čípek, Ladislav Šnajdárek, Lucy Vojtová, Martin Vrbka, Ivan Křupka, Martin Hartl

**Affiliations:** † Department of Tribology, Faculty of Mechanical Engineering, 376202 Brno University of Technology, Technická 2896/2, 616 69 Brno, Czech Republic; ‡ Advanced Biomaterials, Central European Institute of Technology, Brno University of Technology, Purkyňova 656/123, 612 00 Brno, Czech Republic; § Materials Research Center, Faculty of Chemistry, Brno University of Technology, Purkyňova 464/118, 612 00 Brno, Czech Republic; △ Centre of Polymer Systems, University Institute, Tomas Bata University in Zlin, 760 01 Zlin, Czech Republic

## Abstract

This study compares the biotribological and structural behavior of PVA and pHEMA hydrogels under conditions simulating the cartilage environment to understand the lubrication mechanisms. PVA samples exhibited very low apparent friction coefficients and high-water uptake due to their hydrophilic, hydroxyl-rich network. In contrast, pHEMA hydrogels showed higher friction but substantially enhanced wear resistance, particularly under extended sliding against rough counterfaces. While PVA offers excellent lubrication performance, its wear stability remains limited. On the other hand, the low wear observed in pHEMA—despite its higher friction—suggests strong structural resilience, making it a promising platform for further tailoring toward cartilage-mimicking applications. The results highlight the importance of balancing interfacial lubrication and mechanical durability when designing hydrogel-based cartilage replacements.

## Graphical abstract

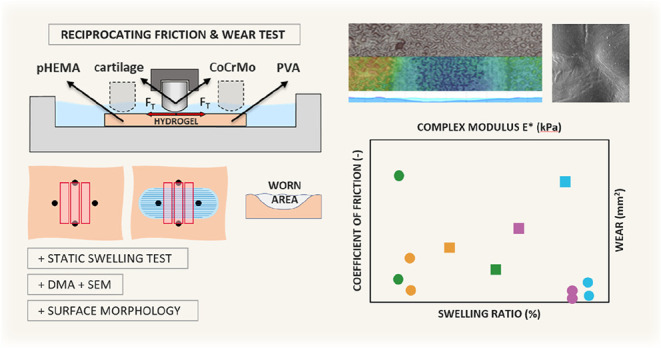

## 1 Introduction

Hydrogels have emerged as a promising class of biomaterials for cartilage replacement due to their high-water content, biocompatibility, and mechanical properties.^1^ The ability of hydrogels to mimic the extracellular matrix of native cartilage makes them suitable candidates for tunable tissue engineering applications^2^ Additionally, their viscoelastic properties allow them to absorb mechanical loads while maintaining a hydrated environment, which is essential for proper cartilage function. However, challenges remain in optimizing their biochemical behavior, mechanical strength, durability, and lubrication properties to withstand the high loads and shear stresses experienced in articulating joints.^3^ Moreover, the degradation behavior and long-term stability of hydrogel-based implants require further study to ensure their viability as long-term solutions in clinical applications.^4^

Current studies on hydrogel-based cartilage repair/replacement focus on enhancing their mechanical properties through cross-linking strategies, composite formulations, and reinforcement with nano- or microparticles to improve load-bearing capacity. Recent advances follow three converging routes: (i) cross-linking strategies to elevate toughness while preserving high water content,^5,6^ (ii) composite and bilayer constructs that couple a lubricious surface with a mechanically robust sublayer or integrate hydrogels with porous backings,^6,7^ and (iii) nanocomposite reinforcement to boost strength, fatigue resistance and adhesion under load.^8,9^ These directions are well documented in recent experimental studies and state-of-the-art reviews on cartilage-mimicking hydrogels.^10,11^

Synthetic hydrogels such as poly­(vinyl alcohol) (PVA) and poly­(2-hydroxyethyl methacrylate) (pHEMA) have been investigated for their structural integrity and ability to retain moisture, which is critical for lubrication in joint applications.^1,4^ Additionally, biotribological evaluations have demonstrated that the lubrication and wear resistance of hydrogels depend on their surface roughness, polymer network architecture, and the presence of synovial fluid components.^12^ Studies have also explored the impact of different fabrication techniques on hydrogel performance.^1^ One key challenge for hydrogel formulations is achieving sufficient mechanical strength while maintaining flexibility. Studies have shown that reinforcing hydrogels with bacterial cellulose-poly­(vinyl alcohol) (BC-PVA) networks significantly enhances their mechanical properties, making them more comparable to native cartilage.^13^ Furthermore, double-network hydrogels incorporating poly­(vinyl alcohol) (PVA) and poly­(2-acrylamido-2-methyl-1-propanesulfonic acid sodium salt) (PAMPS) have been engineered to achieve cartilage-equivalent mechanical robustness.^2^ Additionally, investigations indicate that integrating bioactive molecules, such as growth factors or lubricating proteins, can enhance cellular interactions and further optimize hydrogel performance for tissue engineering applications.^3^ However, balancing these mechanical enhancements with the material’s viscoelasticity remains a crucial aspect of ongoing research. Compared to this, the second pHEMA hydrogel is a biocompatible and cytocompatible polymer with minimal immune response, making it suitable for various biomedical uses. It has been applied in bone regeneration,^14^ wound healing,^15^ cancer therapy,^16^ and ophthalmic devices.^17–19^ Moreover, pHEMA-based hydrogels demonstrate outstanding flexibility, transparency, mechanical strength, and biocompatibility. A major advantage lies in their tunable mechanical and optical properties, which can be readily adjusted by modifying key factors such as the cross-linking method or monomer concentration. Thanks to its favorable mechanical and biological characteristics, pHEMA is also considered a promising material for cartilage replacements.^20,21^

The biotribological performance of hydrogels is influenced by multiple factors, including surface roughness, asperity size, and the interaction between polymer networks and synovial fluid constituents.^22^ Studies have shown that hydrogels with microtextured surfaces can significantly reduce friction under physiological conditions, mimicking the low-friction properties of native cartilage.^23^ Furthermore, hydration lubrication mechanisms play a crucial role in minimizing wear and ensuring long-term functionality, with recent advances highlighting the synergistic effects of surface-bound lubricating molecules and hydrogel elasticity.^3^ Another crucial aspect of hydrogel lubrication is the role of interfacial water layers, which can form a protective barrier against mechanical wear. Recent findings suggest that hydrogels with high hydrophilicity and optimized cross-linking densities exhibit improved boundary lubrication, reducing friction coefficients to levels comparable with native cartilage.^24^ Additionally, studies have investigated polymer brush coatings, such as zwitterionic PMPC layers, which exhibit extremely low friction coefficients due to their hydration lubrication effect.^3^ Furthermore, adaptive lubrication mechanisms in hydrogels, influenced by the interaction of polymer structures with synovial fluid components, have been reported to enhance wear resistance.^4^

Despite significant advancements, there remains a need to develop hydrogel formulations that offer a balance between mechanical robustness and superior lubrication properties. The ability to fine-tune hydrogel properties through polymer composition, cross-linking strategies, and surface modifications remains a critical area of exploration.^25^ Additionally, assessing the mechanical behavior and structural stability of hydrogel-based cartilage replacements under load-bearing conditions is crucial for their effective application.^4^ This study aims to evaluate the biochemical, mechanical, and biotribological performance of standardized and optimized hydrogel systems, formulated according to previously reported protocols^20,26^ but prepared under unified, precisely controlled conditions to minimize variability. Such an approach enables a rigorous comparative assessment of their structure–property relationships under cartilage-mimicking environments. By systematically analyzing the effects of polymer composition and biotribological interactions, we seek to advance the design of hydrogel-based materials that closely replicate the functional properties of natural cartilage. This study aims to bridge the gap between fundamental hydrogel science and the engineering of next-generation cartilage-mimicking materials by elucidating the interplay between structure, chemistry, and tribological performance.

## 2 Materials and Methods

### 2.1 Synthesis of pHEMA Hydrogel Samples

For the synthesis of both types of pHEMA hydrogels, 2-hydroxyethyl methacrylate (HEMA, monomer), 2,2-dimethoxy-2-phenylacetophenone (DMPA, photoinitiator), and ethylene glycol dimethacrylate (EGDMA, cross-linker) were obtained from Sigma-Aldrich (Germany) and Acros Organics (Thermo Fisher Scientific, U.K.). Milli-Q Type 1 water (ISO 3696) was produced using a Millipore purification system (Milli-Q Academic, France). Hyaluronic acid (HA) with a molecular weight of 820–1,020 kg mol^–1^ for the preparation of simplified model synovial fluid (SMSF) was purchased from Contipro a.s. (Czech Republic). A high-intensity UV lamp with a wavelength of 365 nm and 18 W output was obtained from Eurolite (Germany).

pHEMA hydrogels were synthesized with minor modification according to^17^ via UV-initiated free-radical polymerization of HEMA (60% w/w), EGDMA (1% w/w), and DMPA (0.5% w/w) in Milli-Q water, under nitrogen (labeled pHEMA N_2_) or ambient atmosphere (23 °C, 28% RH). The mixture was cast into fixed-volume molds on tribological glass and irradiated with a 365 nm, 18 W UV lamp (10 cm distance) for 20 min. More detailed information regarding pHEMA synthesis may be found in our previous study.^20^

### 2.2 Preparation of PVA Hydrogel Samples

PVA hydrogel samples were prepared using a standardized multistep protocol based on thermal dissolution of PVA and subsequent physical cross-linking. The PVA solution was formulated by dissolving 15 wt % of poly­(vinyl alcohol) powder (Kuraray Co., Ltd.; degree of polymerization: 1700; saponification: 98–99 mol %) in distilled water. Two identical batches were stirred at 500 rpm while the PVA powder was added slowly to avoid agglomeration. After initial homogenization, the closed vessels containing the solution were heated in a water bath setup to maintain the solution temperature between 90 and 95 °C. Stirring was continued for 2–3 h with gradually decreasing revolutions (from 300 rpm to ∼60–80 rpm) to accommodate increasing viscosity. The resulting transparent solution was cooled to room temperature for 2 h under continued mild stirring (120 rpm). After surface film removal, 30 g of the PVA solution was cast into polystyrene dishes (9 cm diameter) for hydrogel fabrication. Air bubbles were removed manually using a pipet.

The CP06 hydrogel samples were prepared via a combined cast-drying and freeze–thaw method (CP process). Following solution casting, dishes were sealed and subjected to a single freeze–thaw cycle (−20 °C for 8 h → 4 °C for 16 h), followed by high-temperature drying under controlled conditions (60 °C, 80% RH, 6 h). The partially dried samples were then subjected to a second freeze–thaw cycle of the same parameters and finally exposed to low-temperature drying (8 °C, 50% RH) until water content fell below 12%, typically after 4–5 days. Dried gels were immersed in distilled water (1 L per sample) and left to swell for 3 days. The bottom surface (i.e., the one in contact with the dish) was marked and used as the sliding surface for subsequent tribological testing.

The LM hydrogels were fabricated as a bilayer system, consisting of a freeze–thaw (FT) base layer and a cast-dried (CD) top layer. First, a dose of PVA solution was subjected to four FT cycles (−20 °C for 8 h, 4 °C for 16 h each). A second layer (CD) was then cast onto the FT base and dried at 20 °C and 50% RH. The drying was continued until residual water content was below 12%, which typically required ∼3 days. After swelling in distilled water for 3 days, the upper surface of the laminar construct (the one exposed to air during drying) was used as the sliding surface. All samples were stored in distilled water at 4 °C prior to characterization. Further details about the hydrogel preparation may be found in ref 26.

### 2.3 Characterization of Materials

#### 2.3.1 Static Swelling Test

PVA hydrogel samples in the form of 10 mm compact discs were left to dry in a laboratory oven (Ecocell 111, Thermo Fisher Scientific, Czech Republic) at a temperature of 50 °C until they reached a constant weight. The average thickness of dried PVA CP06 (transparent) and layered PVA (opaque) was 1.38 ± 0.4 and 1.82 ± 0.4 mm, respectively. A SMSF solution, consisting of 0.5% w/w hyaluronic acid (HA) in Milli-Q water, was prepared and used for the swelling experiments. The hydrogel samples were immersed in the model synovial fluid and incubated at 37 °C. After the selected time points (0.5, 1, 2, 3, 4, 5, 6, 24, 26, 29, 48, and 72 h) of incubation, the samples were gently wiped with a lint-free cloth to remove surface moisture, and water uptake was quantified gravimetrically. Each experiment was conducted in quintuplicate (*n* = 5), and the swelling ratio of the hydrogels was calculated according to eq 1.

1 $$swelling\;ratio\;\left(\%\right)=\frac{W_{1}-W_{0}}{W_{0}}\cdot 100$$

where *W*_0_ is the initial weight of the dried sample and *W*_1_ is the weight of the swollen sample at the defined time interval.

A first-order swelling kinetic model was used to fit the experimental data and to calculate the swelling parameters according to eq 2 ^27,28^

2 $$\frac{dS}{dt}=k_{1}\cdot \left(S_{\max}-S\right)$$

where *k*_1_ is the rate constant for first-order swelling kinetics, *S* is the degree of swelling at a specific time point, and *S*_max_ is the degree of swelling at equilibrium.

The first-order (*k*_1_) swelling constant and *S*_max_ were calculated by fitting the experimental data to the model in Excel using the function “Solver” with the parameters set to be larger than or equal to zero.

#### 2.3.2 Dynamic Mechanical Analysis

Compression tests were performed on the swollen hydrogels (for 72 h) using dynamic mechanical analysis (DMA) on the DHR-2 instrument (TA Instruments), equipped with cross-hatched plate-to-plate geometry (diameter 20 mm). Tests were conducted in time sweep mode at constant conditions: temperature of 25 °C, frequency of 1 Hz, and displacement amplitude of 12 μm. This corresponded to ≤1% compressive strain, ensuring the measurements remained within the linear-viscoelastic region while applying the same absolute deformation to every specimen regardless of height. For the compression tests, the samples were shaped into circles with a precise cutting tool (standardized punch, diameter 20 mm). Prior to measurement, each sample was compressed to a normal force of 2.5 N and allowed to relax for 2 min. This in-house protocol was designed in accordance with the general guidelines for axial DMA testing of soft hydrogels, as described by TA Instruments (Application Note EF034).^29^

#### 2.3.3 Surface Morphology

The surface features of dried PVA hydrogel samples were examined using scanning electron microscopy (SEM). Prior to SEM analysis, the samples, dried to constant weight in a laboratory oven (Ecocell 111) at 50 °C, were coated with a 15 nm-thick gold layer using a Leica EM ACE600 coater (Leica, Germany). Imaging was performed using a MIRA3-XMU microscope (Tescan, Czech Republic) at appropriate magnifications and a working distance, with an accelerating voltage of 5 kV. Images were processed using ImageJ 2 software (National Institutes of Health, USA).

### 2.4 Pin-on-Plate Friction and Wear Tests

A commercial tribometer (Bruker UMT TriboLab) was employed to perform reciprocating sliding experiments. This device allows for multiple test configurations suitable for studying tribological behavior. In this study, two setups were used: a pin-on-plate configuration with CoCrMo-on-PVA and cartilage-on-PVA contact pairs. Details of the experimental are provided in Table 1. A reciprocating pin-on-plate configuration was selected to ensure precise control of kinematic parameters (load, stroke, velocity) and to facilitate direct, comparative evaluation of the hydrogel systems under identical lubrication conditions. This configuration has been routinely employed in previous tribological studies from our laboratory, focusing on cartilage interactions.^30–32^ Similar reciprocating pin-on-flat or pin-on-disc geometries have also been used by other groups investigating hydrogel/cartilage-surrogate tribology.^33–37^ This configuration supports mechanism-oriented, comparative evaluation of friction and wear. Together, these studies support the relevance and reproducibility of this approach for comparative biotribological evaluations.

**Table 1:** Experimental Conditions of Friction and Wear Tests

	friction test	wear test
material	PVA (CP06, LM) pHEMA (air, nitrogen)	
load	5 N	
stroke	12 mm	
lubricant	model synovial fluid	
speed	10 mm·s^–1^	
pin (ø)	9.7 mm	
pin material	cartilage	CoCrMo
no. of cycles	750	7500
duration	30 min	5 h
repetition	5 times	3 times

Friction measurements were obtained using a load cell module integrated into the slider mechanism. The module included a biaxial load cell and sensors that maintained a constant normal force of 5 N and recorded the corresponding tangential force. The load cell had a maximum capacity of 50 N. The slider produced a consistent linear motion driven by a stepper motor (see Figure 1). Data collected at the stroke reversals, where the sliding speed dropped below the target value, were excluded from the analysis.

**Figure 1:**
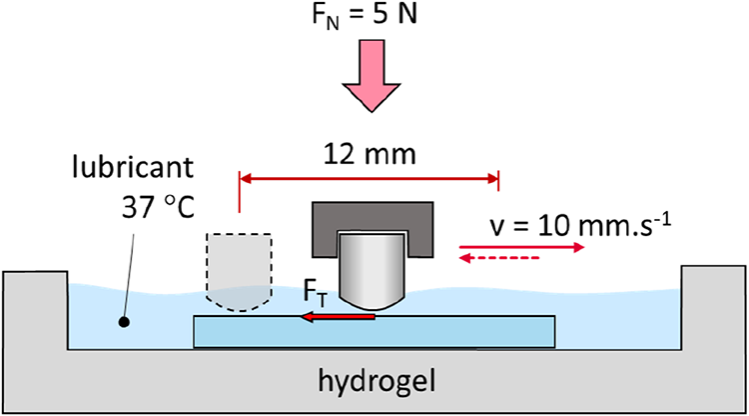
Tribometer experimental setup.

As outlined in Table 1, two sets of tests were performed: friction tests and wear tests. These differed in both the type of sliding pin used and the test duration. To ensure statistical robustness, each test was repeated five times with cartilage and three times with the CoCrMo pin. Based on initial trials, where negligible wear was observed on both cartilage and polished CoCrMo surfaces, the CoCrMo pin was intentionally roughened to an arithmetical mean height of Sa = 530 nm for the main wear experiment.

All experiments were performed at 37 ± 1 °C to simulate physiological joint temperature. The temperature was maintained by a thermostatically controlled heating stage integrated in the tribometer, equipped with a feedback loop and resistive heating elements positioned below the lubricant chamber. The lubricant (model synovial fluid) and contact zone were continuously monitored using an external thermometer immersed in the fluid to verify temperature stability. Prior to testing, the lubricant was preheated, and the experiment was initiated only after thermal equilibrium (37 °C ± 1 °C) was reached within the test chamber. This protocol, routinely used in our laboratory, ensures consistent measurement conditions and minimizes variations due to thermal swelling or viscosity changes of the hydrogel samples.

#### 2.4.1 Contact Conditions

The pin specimen consisted of a bovine articular cartilage cap with a spherical end and a flat hydrogel plate. The radius of curvature of the cartilage pins was quantified using an optical scanning system applied to 10 freshly prepared cartilage caps before punching. The average measured radius was 29.9 mm, which is in very good agreement with previously published geometric data for bovine femoral heads.^38^ The measured curvature, therefore, confirms that the prepared cartilage pins faithfully represent the native bovine femoral head geometry used in cartilage–surrogate tribology. The mechanical properties are summarized in Table 2. The properties of the PVA-based hydrogels were determined by the authors’ team in a previous study.^26^ The properties of cartilage were obtained from the literature.^38^ Using a nonlinear finite element contact analysis accounting for the variable stiffness of the interacting materials (bone, cartilage, and hydrogel). The calculated mean contact pressure under a 5 N load was calculated to be between 0.17 and 0.18 MPa. Since the compressive modulus of the pHEMA hydrogel could not be reliably determined, the mean contact pressure was estimated from the optically observed contact area under the transparent specimen (*d* = 6.7 mm), resulting in an estimated contact pressure of approximately 0.14 MPa. The obtained contact pressures fall within the lower portion of the physiological range commonly reported for articular cartilage under gait and low-impact loading conditions (0.1–2 MPa), see.^39–41^ The elastic moduli listed in Table 2 (used in the FE contact model and load-partitioning estimates) are quasi-static values obtained from our prior work or literature, whereas DMA reports the small-strain dynamic complex modulus E* at 1 Hz (Section 2.3). Because these quantities probe different deformation modes and time-scales, they are not directly comparable; we therefore use elastic moduli for contact stress estimation and complex moduli to discuss frequency-dependent stiffness trends.

**Table 2:** Mechanical Properties of the Test Specimens

	PVA CP06	PVA LM	pHEMA-N_2_	pHEMA-air	cartilage
compressive/tensile modulus *E* (MPa)	0.51^a^,^26^	0.63^a^,^26^	0.92^b^,^20^	0.82^b^,^20^	1.86 ^38^
Poisson’s ratio μ (−)	0.45	0.45	0.45	0.45	0.45
measured contact zone diameter (mm)			6.7	6.8	
estimated mean contact pressure *p* (MPa)	0.17	0.18	0.14	0.14	

a Compressive modulus used in the nonlinear finite element simulations.

b Tensile modulus not considered in the calculations; for these samples, the mean contact pressure was estimated from the optically determined apparent contact area.

#### 2.4.2 Cartilage Preparation and Conditioning

Bovine cartilage was harvested from adult animals (18–24 months) within 5 h after slaughter, rinsed with PBS, and stored in PBS at −22 °C. Before testing, samples were thawed at ambient temperature when immersed in PBS, and visually inspected. To minimize the variance in the radius of curvature of the cartilage caps, the pins were excised from the peripheral region of the femoral head, where the surface curvature is nearly uniform and readily measurable. Specimens with any sign of surface irregularity or uncertainty in integrity were excluded. First, larger cartilage caps were sectioned from the peripheral zone of the femoral head using a saw. Subsequently, individual cartilage pins were punched out from the central area of each trimmed cap using a custom stainless-steel punch. This procedure is routinely applied in our laboratory and provides consistent geometry and surface quality of the cartilage pins. Specimens showing any surface irregularities or compromised integrity were excluded from further testing. The entire procedure, along with the actual appearance of representative cartilage pin, is illustrated in Figure 2.

**Figure 2:**
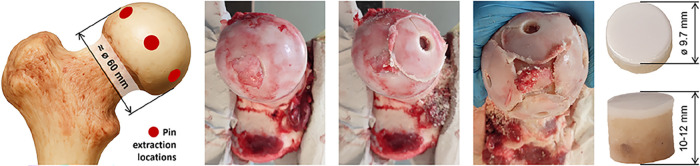
Procedure for cartilage pin extraction.

#### 2.4.3 Model Synovial Fluid

The lubricant used in all experiments was a *model synovial fluid* developed in-house in collaboration with the biochemists from University Hospital Olomouc. The formulation was derived from extensive biochemical analyses of synovial fluid samples obtained from patients without hip joint replacement. This approach ensured physiologically relevant concentrations of proteins, hyaluronic acid, and phospholipids, representative of realistic lubrication conditions. The complete composition of the model synovial fluid is listed in Table 3, and the same formulation has been routinely employed in previous tribological studies within our laboratory.

**Table 3:** Composition of a Simplified Model Synovial Fluid^a^

	albumin	γ-globulin	hyaluronic acid	phospholipids
concentration (mg·mL^–1^)	20	3.6	2.5	0.15

a Albumin (Sigma-Aldrich A7030); γ-globulin (Sigma-Aldrich G5009); phospholipids (Sigma-Aldrich P3782).

### 2.5 Surface Topography Analysis

Additionally, both untested and worn samples were examined using optical scanning microscopy (OSM, Keyence VHX 7000). The scanned surfaces were assessed visually and by measuring cross-sectional wear area. Optical evaluation focused only on samples that had been subjected to sliding with the roughened CoCrMo pin. The observation procedure is illustrated in Figure 3. For each sliding track, three images (2 × 10 mm^2^) were captured at a predefined location, marked with a crosshair using an alcohol-based pen. Images were taken both before and after the test to enable area loss assessment, as shown on the right side of Figure 3.

**Figure 3:**
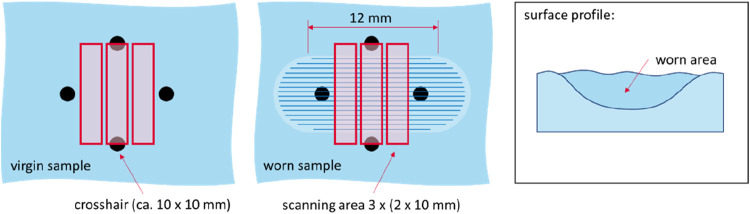
Left: virgin sample; middle: worn sample; right: illustration of the data obtained from scanned areas.

## 3 Results

### 3.1 Swelling Capacity, DMA and Surface Morphology

The swelling behavior of two types of PVA hydrogels, specifically a transparent monolayered PVA CP06 and an opaque, layered PVA, was investigated in a SMSF containing 0.5% w/v hyaluronic acid (HA) in Milli-Q water. The data were subsequently fitted to first-order kinetic equations, and the first-order swelling constant (*k*_1_) along with the degree of swelling at equilibrium (*S*_max_) were calculated (Table 4) using the Excel “Solver” function.

**Table 4:** Calculated First-Order Swelling Rate Constant *k*_1_, Correlation Coefficient *R* ^2^, and the Predicted Degree of Swelling at Equilibrium *S*_max_ of the PVA CP06 and Layered PVA LM^a^

	PVA CP06	PVA LM	pHEMA N_2_ ^20^	pHEMA air^20^
*k*_1_ (h^–1^)	0.26 ± 0.01	0.16 ± 0.01	1.29 ± 0.54	1.04 ± 0.29
*R* ^2^ (−)	0.99 ± 0.00	0.99 ± 0.01	0.98 ± 0.01	0.97 ± 0.02
*S*_max_ (%)	235.57 ± 2.94	219.91 ± 4.91	53.61 ± 2.40	60.32 ± 9.45

a The data for the pHEMA hydrogels synthesized under varying atmospheres in a SMSF were retrieved from our previous publication^20^.

The PVA and pHEMA hydrogels exhibit distinct swelling behaviors in environments containing hyaluronic acid, which are influenced by their material properties and mechanisms of interaction with the SMSF components. As illustrated in Figure 4, PVA hydrogels (represented by the blue and purple curves) demonstrate a high swelling capacity in hyaluronic acid-containing environments.

**Figure 4:**
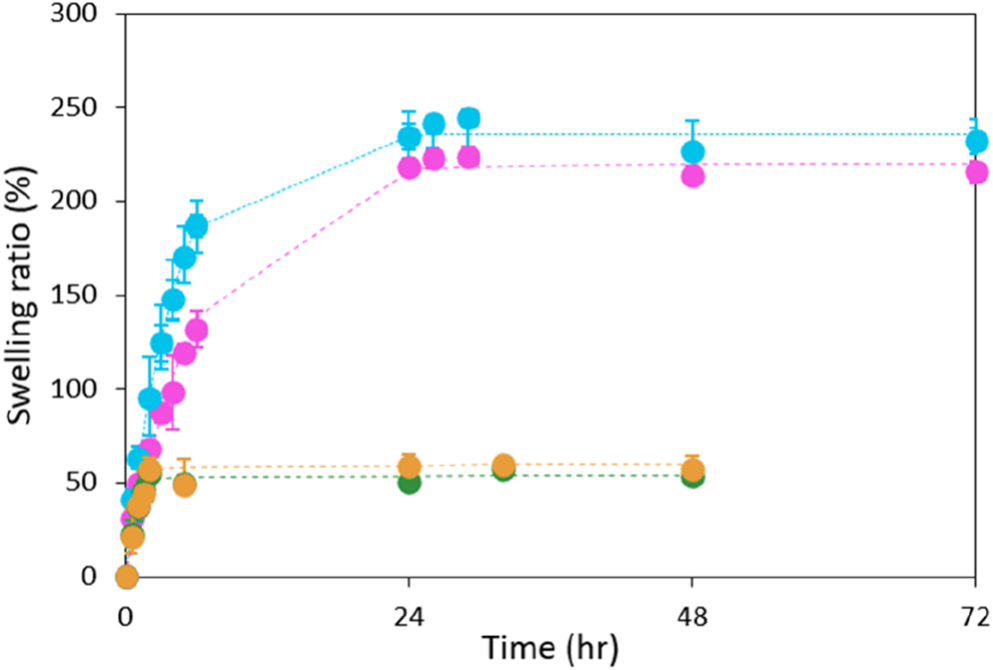
Swelling ratios of the two distinct PVA hydrogels compared to the pHEMA hydrogels prepared under nitrogen and laboratory atmospheres in a SMSF. The time points were fitted using a first-order swelling kinetic model. The data for the pHEMA N_2_ and pHEMA air were retracted from our recent publication.^20^

PVA hydrogels prepared via the freeze–thaw method develop a macroporous network that enhances the absorption of aqueous solvents.^42^ In our study, both hydrogel types exhibited rapid swelling,^43^ reaching equilibrium after 24 h of submersion in SMSF, after which their water content remained stable. The equilibrium swelling ratios for PVA LM and CP06 were 218.1 ± 4.1 and 234.5 ± 7.0%, respectively. The slightly higher swelling ratio of the single-layered CP06 hydrogel can be attributed to its structure, consisting of a single PVA layer subjected to two freeze–thaw and two cast-drying cycles. In contrast, the PVA LM hydrogel is composed of a layered structure combining four freeze–thaw cycles with cast-drying, which contributes to its comparatively lower swelling. Freeze–thaw promotes the formation of a macroporous structure in the hydrogel, further enhancing water uptake and swelling rate.^44^ On the other hand, cast-drying results in a denser network due to tighter hydrogen bonding and reduced porosity, which limits the swelling capacity.^45,46^ Additionally, the formation of extra hydrogen bonds during cast-drying further restricts the rehydration potential of the hydrogel in simulated body fluids. It is also notable that an increased number of freeze–thaw cycles leads to reduced swelling in freeze-dried PVA hydrogels.^47^ Thus, both the number of freeze–thaw cycles and the inclusion of cast-dried PVA layers influenced the swelling behavior observed in the PVA LM hydrogels.

In comparison, the pHEMA hydrogel exhibited a significantly lower swelling ratio, primarily due to its denser and more rigid polymer network formed by chemical cross-linking. This structure contains fewer accessible hydrophilic groups, which delays and limits the overall water absorption.^48,49^ Although the chemical structure of pHEMA retains some hydrophilicity, it does not match the swelling potential of PVA, resulting in restricted swelling in water and biological fluids. In contrast, PVA hydrogels, composed of long polymer chains uniformly distributed with hydroxyl groups, form a flexible and highly hydrophilic network. This allows extensive hydrogen bonding with water molecules, enabling greater swelling capacity.^50,51^ As we reported in our previous study,^20^ bovine articular cartilage swells rapidly in SMSF, reaching a swelling ratio of 222.8 ± 43.3% within several hours. Therefore, in terms of swelling behavior, the layered PVA LM hydrogel presents a promising candidate for mimicking the response of bovine articular cartilage in simulated model synovial fluid.

#### 3.1.1 Dynamic Mechanical Analysis

DMA was used to obtain a more detailed understanding of the mechanical behavior of the material under dynamic loading, which is closely related to its tribological properties. Time-sweep compression tests (25 °C, 1 Hz, 12 μm) showed that all hydrogels were elastic-dominated (*E*′ ≫ *E*″). The two PVA hydrogels, CP-06 and LM, exhibited the highest initial stiffness (≈110 kPa and ≈90 kPa, respectively) and remained essentially unchanged, indicating excellent mechanical stability. In contrast, the pHEMA hydrogels (N_2_ and air) displayed a gradual increase in both moduli, a behavior typical of dehydration-driven densification reported for pHEMA networks.^52^ Figure 5 displays a complex modulus E* calculated following eq 3.

3 $$E^{*}=\sqrt{E^{\prime 2}+E^{\prime \prime 2}}$$

where *E*′ refers to storage modulus (MPa) and *E*″ is the loss modulus (MPa).

**Figure 5:**
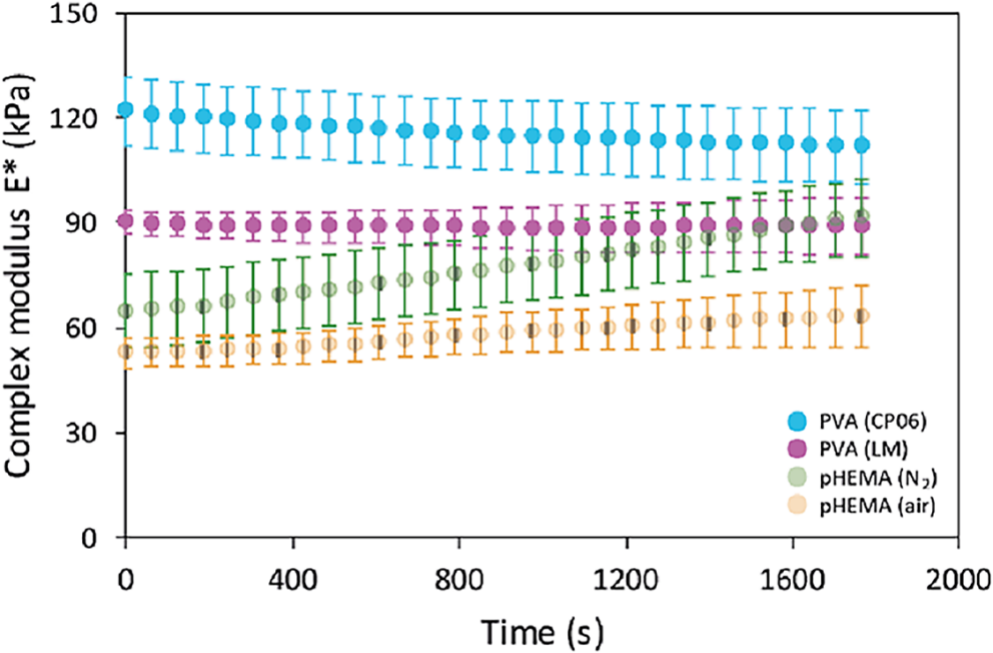
Development of the complex modulus during time-sweep tests for four hydrogel systems at 25 °C, 1 Hz, ≤1% strain. *The semitransparent data points (pHEMA hydrogels) correspond to results that were previously published as Supporting Information in our earlier study.^20^

Figure 6 shows an example of the morphology of all hydrogel samples observed under SEM. On close examination, it can be noticed that the surface of the pHEMA-air sample has an unmistakable wrinkled surface (Figure 6C), while PVA CP06 (Figure 6A) shows fine wrinkling. On the other hand, the PVA LM sample (Figure 6B) already shows a smooth surface compared to PVA CP06. A similar smooth surface morphology of the pure PVA sample was demonstrated by Jayaramudu et al.^53^ The pHEMA N_2_ sample exhibits a smooth and homogeneous morphology, as evidenced by its uniform surface texture (Figure 6D). It should be noted that SEM imaging requires complete drying of the hydrogel, which inevitably alters its true hydrated morphology. Drying collapses internal porosity, suppresses surface swelling features, and may introduce artificial wrinkling, meaning that SEM micrographs should be interpreted qualitatively rather than as direct representations of the hydrated surface. For this reason, surface roughness values reported in this study are based on hydrated-state optical scanning microscopy (Section 3.3), which provides a more relevant quantitative assessment of surface smoothness for tribological interpretation. Future studies will employ hydrated-state imaging using Advanced Environmental SEM (A-ESEM) to directly visualize the microstructure and surface features of both hydrogels without drying artifacts.

**Figure 6:**
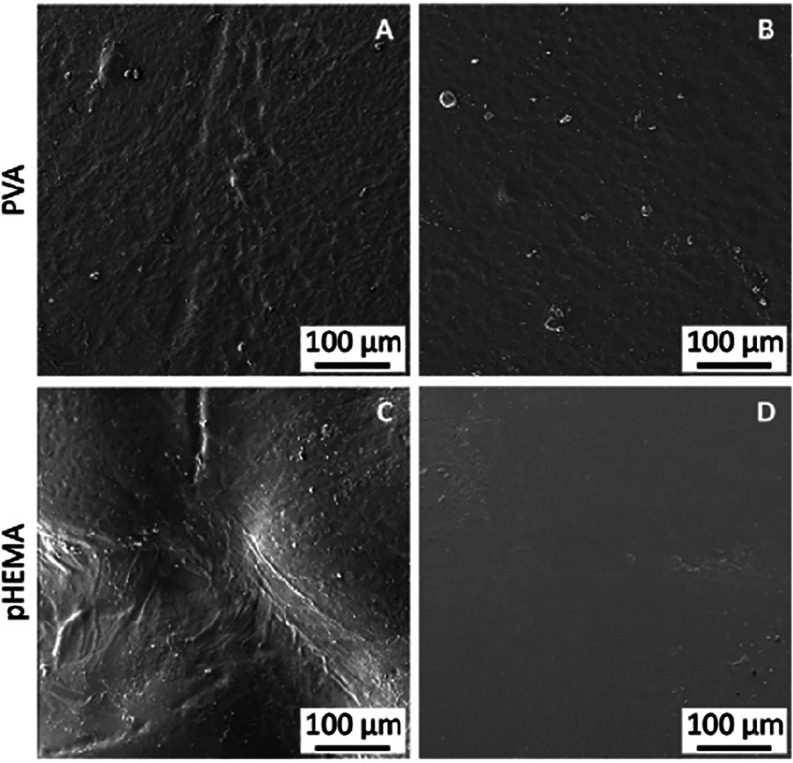
SEM images of dried hydrogel samples: (A) PVA CP06, (B) PVA LM, (C) pHEMA air, and (D) pHEMA N_2_.

### 3.2 Friction Analysis

The sliding experiments against the cartilage pin revealed a pronounced difference in the coefficient of friction (COF) between PVA and pHEMA samples (Figure 7, top). After 750 cycles, the pHEMA-N_2_ sample exhibited a COF of 0.086, while pHEMA-air reached 0.026. In contrast, PVA LM and CP06 demonstrated markedly lower friction values of 0.008 and 0.006, respectively. This very low apparent friction performance is counterbalanced by the difficulty of preserving surface smoothness during PVA fabrication, which remains a critical limitation in material selection. Even minor surface irregularities lead to an increase in COF—though the rise remains negligible compared to pHEMA.

**Figure 7:**
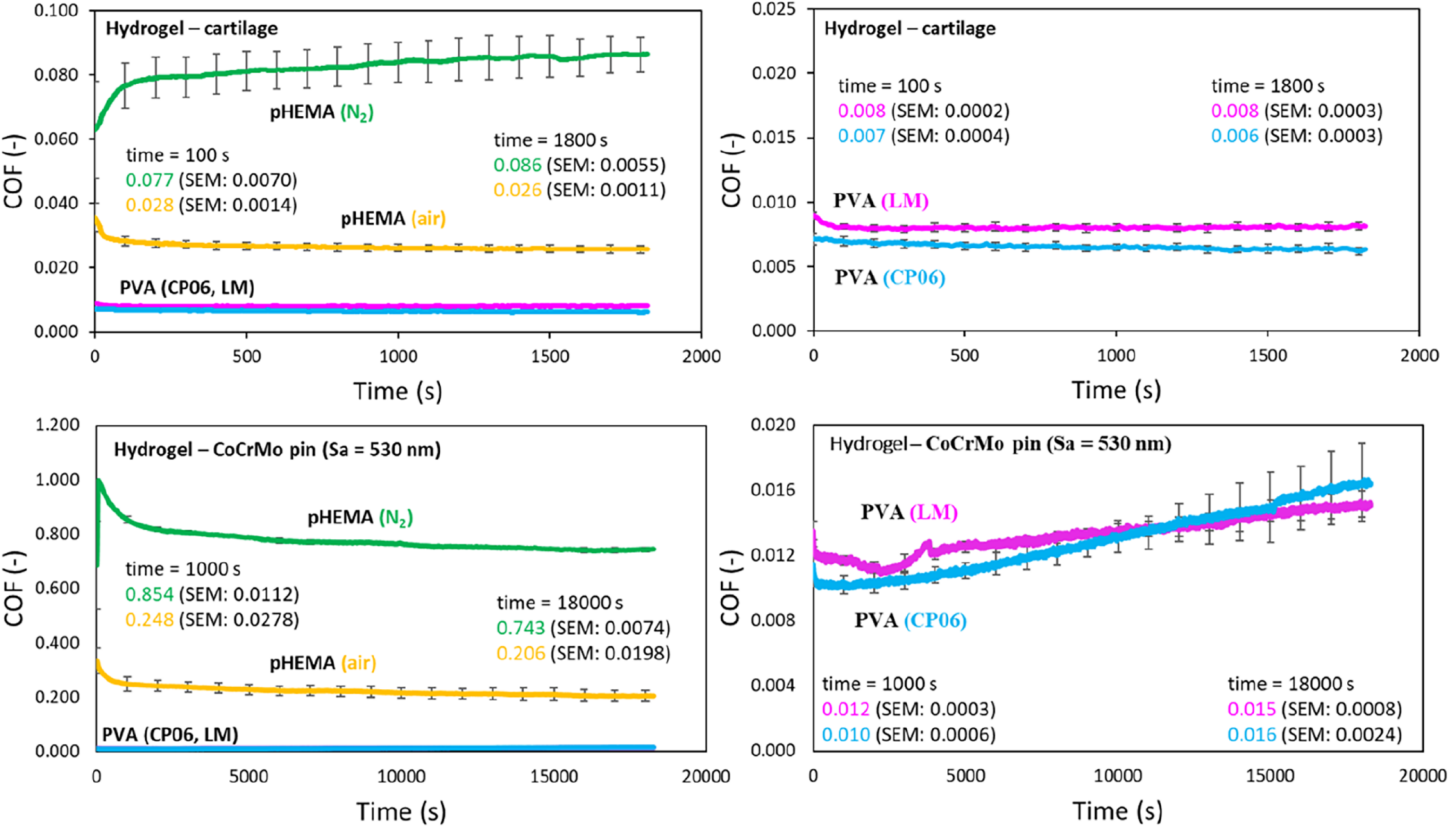
Friction comparison of PVA and pHEMA hydrogels sliding against cartilage pin (top), and roughened CoCrMo pin (bottom).

When the sliding pin was replaced with CoCrMo, the disparity between the hydrogel types became even more evident (Figure 7, bottom). PVA samples maintained low friction levels in the range of 0.010–0.016, whereas pHEMA showed a significant increase in COF—approximately an order of magnitude higher than in the cartilage pin setup. From a purely frictional standpoint, the hydrogels performed in the following order (from best to worst): PVA CP06 ≈ PVA LM ≪ pHEMA-N_2_ < pHEMA-air.

### 3.3 Wear Analysis

The topography and cross-sectional wear area of the hydrogel samples were assessed using OSM. The pHEMA-air samples displayed a visibly wavy surface with an arithmetical mean height of Sa = 9.0 ± 1.4 μm, whereas the pHEMA-N_2_ formed a smoother and more uniform layer exhibiting Sa = 1.2 ± 0.3 μm. In contrast, PVA samples, cross-linked exclusively in air, consistently resulted in smooth surfaces, with PVA CP06 exhibiting Sa = 1.5 ± 0.4 μm and PVA LM Sa = 1.4 ± 0.3 μm.

Although lower wear might be anticipated for PVA based on its lower COF, the experiments showed the opposite trend: the apparent cross-sectional wear area was consistently smaller for pHEMA than for PVA (Figure 8). However, the reliability of these measurements is limited, as hydrogel surfaces experienced partial drying during scanning, and the convex specimen geometry introduces focus and depth artifacts. For this reason, OSM-based wear areas should be interpreted cautiously. To improve reproducibility, all hydrogel samples were firmly adhered to glass plates prior to scanning to suppress macroscopic waviness, and for each hydrogel type, three independent samples were analyzed, with three longitudinal profiles extracted from each (nine measurements per material). Despite the inherent limitations of optical scanning, these replicated measurements displayed consistent qualitative trends across specimens. Therefore, the reported wear values should be regarded as comparative indicators rather than absolute representations of cartilage-mimicking contact conditions. The primary purpose of including these data is to demonstrate the markedly higher structural durability of pHEMA compared with PVA under the applied testing regime. Additional correlation plots between friction, wear, swelling capacity and mechanical stiffness are provided in the Supporting Information file (Section S1 and Figure S1).

**Figure 8:**
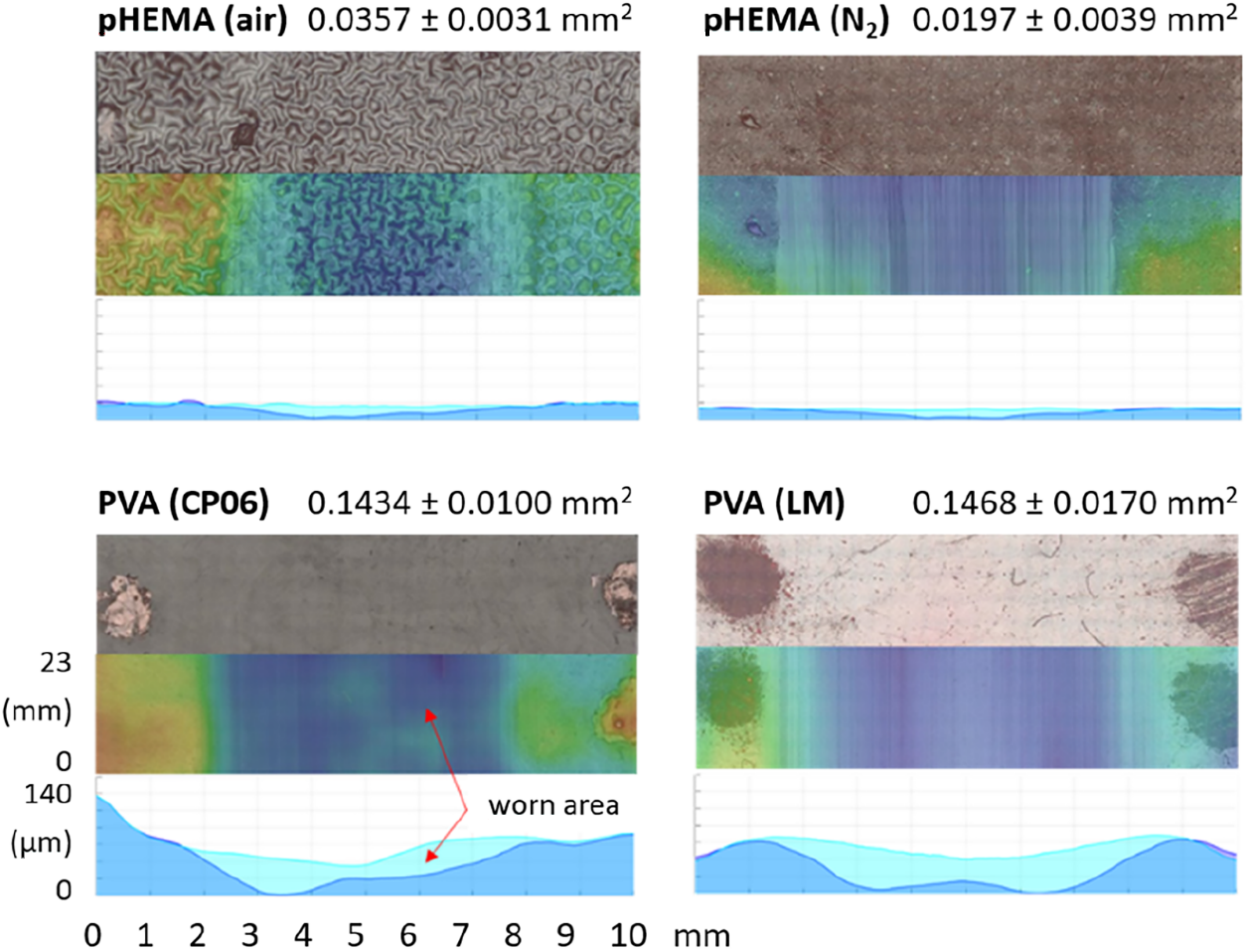
Representative images of scanned hydrogel samples and their cross-sectional wear area ± SEM in mm^2^ after wear test.

## 4 Discussion

To provide context for the tribological findings, it is important to first discuss the load-bearing mechanical performance of the evaluated hydrogels. The materials used in this study were not intended as load-bearing cartilage replacements, but rather as transparent model systems for mechanistic investigation of lubrication and interfacial water transport. Full transparency was required to enable optical monitoring of the contact area and, in future studies, fluorescence-based visualization of hydration and boundary-layer dynamics. This design constraint inherently limits the achievable cross-link density and polymer content, resulting in compressive stiffness values one to 2 orders of magnitude lower than those of native cartilage. Consistent with this, the dynamic compressive stiffness of the tested hydrogels (≈50–120 kPa) is substantially lower than that of human articular cartilage, for which a dynamic complex modulus *E* ^*^ typically slightly exceeds 30 MPa at 1 Hz^54^. Such values are fully in line with those reported for physically cross-linked PVA or pHEMA hydrogels, which generally exhibit dynamic moduli in the tens-to-hundreds of kPa range.^55–57^ Enhancing the elastic modulus would inevitably reduce transparency, illustrating the trade-off between mechanical robustness and optical accessibility that characterizes such model systems. Accordingly, the present hydrogels should be viewed as lubrication-mimicking surrogates rather than load-bearing stiffness replacements. Future developments will focus on reinforced or composite architectures to increase modulus while maintaining favorable tribological behavior.

The lowest friction coefficients of 0.006 and 0.008 were measured for PVA CP06 and PVA LM, respectively, during articulation against a cartilage pin. These values should be interpreted as very low apparent friction under cartilage-mimicking specific laboratory conditions, rather than absolute cartilage-level performance. pHEMA-based hydrogels exhibited approximately 3- to 8-fold higher coefficients of friction. A promising result was observed for pHEMA (air), which showed a stable COF of 0.026 throughout the experiment, comparable to PVA. In contrast, pHEMA polymerized in a nitrogen atmosphere showed lower repeatability and a gradually increasing COF. These results may be explained by the different water content and permeability of the hydrogel samples. As shown in Figure 9, the PVA samples stood out with the highest water content (≈73%) compared to pHEMA hydrogels (N_2_: 54%, air: 61%). It is worth noting that our previous study^20^ reported a coefficient of friction of approximately 0.016 for cartilage-on-cartilage articulation under similar test conditions. This physiological value serves as a useful benchmark for interpreting the present results. While both PVA hydrogels exhibited even lower COF values in this setup, such performance must be balanced against other factors such as wear resistance and long-term durability. The contact analysis revealed that the mean pressure under a 5 N load was between 0.14 and 0.18 MPa, corresponding to the lower physiological range reported for articular cartilage (0.1–2 MPa).^39–41^ The friction values thus reflect the relative response of the hydrogel systems under controlled yet simplified geometry and lubrication conditions, allowing robust comparison but not full replication of joint biomechanics. Quantitative surface analysis showed that the untested pHEMA-N_2_ hydrogel exhibited the lowest arithmetical mean height (Sa = 1.2 ± 0.3 μm), followed by PVA LM (Sa = 1.4 ± 0.3 μm), PVA CP06 (Sa = 1.5 ± 0.4 μm), and pHEMA-air (Sa = 9.0 ± 1.4 μm). These results indicate that surface roughness alone cannot explain the frictional trends observed among the hydrogel systems. The pHEMA-N_2_ hydrogel, despite exhibiting the lowest arithmetical mean height, showed the highest friction, whereas pHEMA-air, with the roughest surface, displayed intermediate friction values between PVA and pHEMA-N_2_. This apparent inconsistency highlights that chemical composition and hydration capacity play a dominant role over topography. PVA’s hydroxyl-rich polymer network promotes the formation of stable hydration layers and effective boundary lubrication, while pHEMA—with a less hydrophilic backbone and reduced capacity for bound water—exhibits weaker surface hydration and thus higher friction.

**Figure 9:**
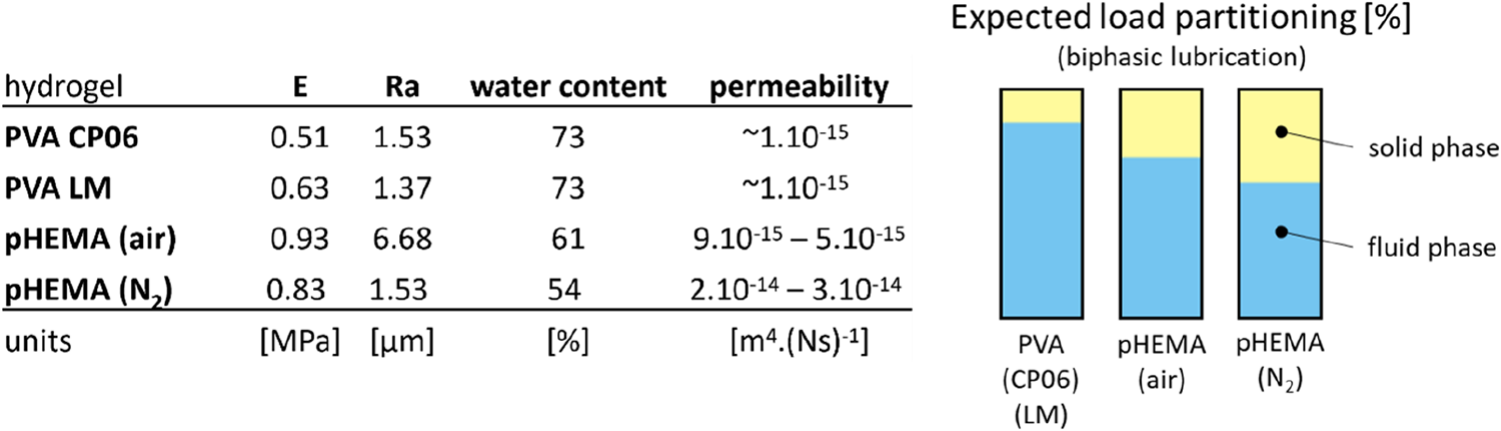
Comparison of properties influencing the tribological properties of hydrogels at the reciprocal test. Note: Raw data comes from virgin samples; the permeability of CP06 and LM is estimated based on ref 58,59 . The expected load partitioning is estimated according to ref 60.

The excellent performance of both PVA samples can be attributed to a high degree of fluid load support and only minimal solid-to-solid contact. Consistent with the water content, the COF trend of the pHEMA samples follows the order CP06 < LM < (air) ≪ (N_2_), see Figure 7. An important factor contributing to good lubrication is the permeability of interstitial water. However, despite its importance, the permeability of cartilage is relatively low (approximately 10^–15^ to 10^–16^ m^2^), which allows for optimal fluid outflow and uptake under load. Higher permeability, as seen in pHEMA (N_2_) or PVA FT^58^ typically results in higher friction. The exceptional lubrication of PVA is likely further supported by highly hydrated surfaces formed by protruding surface polymer chains.^22^ Additionally, the bumpy surface of pHEMA (air) may contribute to the low COF by acting as a lubricant reservoir (enhancing elastohydrodynamic lubrication) and by facilitating additional biphasic squeeze-out, see Figure 10. This unique surface morphology of the pHEMA-air hydrogel may stem from oxygen inhibition during photopolymerization, which is known to interfere with free-radical chain propagation near the polymer–air interface.^61^ As a result, the uppermost layer of the hydrogel exhibits reduced cross-linking density and a more irregular polymer network. This leads to a locally softer and more hydrated region with elevated permeability and enhanced fluid mobility. The presence of this surface-modified layer not only lowers the coefficient of friction due to improved hydration lubrication but also facilitates rapid fluid exchange near the interface.^62^ Despite the low permeability of the bulk material, this localized permeability allows for temporary fluid pressurization during loading while enabling the regeneration of the lubrication layer between cycles, thereby sustaining low friction throughout repeated articulation.

**Figure 10:**
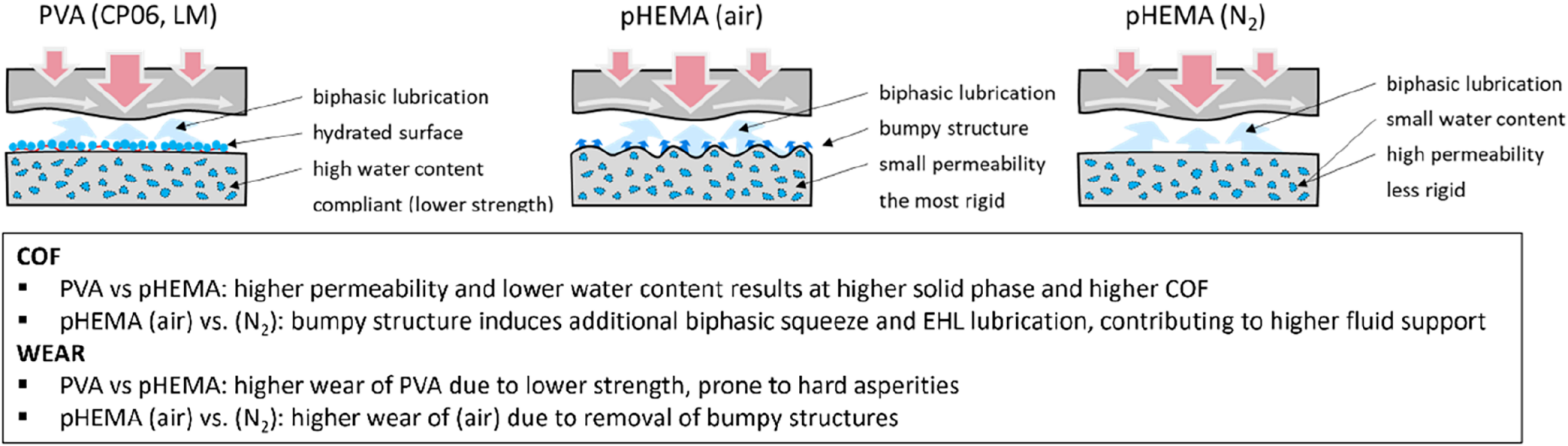
Explanation of long-term wear test results with CoCrMo pin.

Beyond physical surface features, the observed differences in friction performance between PVA and pHEMA hydrogels may also be attributed to their inherent chemical structure and resulting affinity for water. PVA contains numerous hydroxyl (−OH) groups along its polymer backbone, readily forming hydrogen bonds with surrounding water molecules. This promotes the formation of a stable hydration layer at the hydrogel surface, facilitating low-friction sliding under aqueous conditions. In contrast, pHEMA possesses only a single −OH group per monomer and a predominantly hydrophobic methacrylate backbone, resulting in a lower density of hydrogen bonding interactions. Consequently, pHEMA hydrogels exhibit weaker surface hydration and less effective boundary lubrication, which may contribute to their consistently higher coefficients of friction. These findings are consistent with the swelling data, where PVA samples demonstrated significantly greater water uptake, reflecting their stronger hydrophilic character.

The interplay between mechanical stiffness and frictional performance appears to differ substantially between PVA and pHEMA hydrogels. In PVA, increased compressive modulus has been correlated with reduced friction coefficients,^63^ suggesting that enhanced structural integrity contributes to better load support and fluid retention during articulation. In contrast, pHEMA hydrogels tend to achieve lower friction when the surface is soft and swollen, implying that surface hydration and chemistry dominate lubrication behavior rather than stiffness alone.^62^ These observations highlight that distinct lubrication mechanisms are at play depending on the polymer’s structure and interaction with the surrounding medium.

During testing with the cartilage pin, no visible wear was observed on the hydrogel samples. Therefore, a roughened CoCrMo pin (Sa = 530 nm) was used as a counter surface. The results showed the same sample ranking based on their COF. The PVA samples maintained approximately the same friction level as in the cartilage test (≈0.015), slightly increasing as wear progressed. A major difference was observed for the pHEMA samples, where the COF increased by up to 10 times (e.g., air: 0.026 → 0.206) compared to the cartilage test, see Figure 7. The COF trend of the pHEMA samples remained either stable or slightly decreasing over time. Interestingly, the lowest wear was not observed in the PVA samples—despite their lowest COF—but in the pHEMA samples, see Figure 8. This phenomenon is likely due to the higher elastic modulus, which correlates with greater material strength and thus reduced susceptibility to damage from hard asperities during solid-to-solid contact (e.g., CP06 = 0.51 MPa, pHEMA (air) = 0.93 MPa). Surprisingly, the lowest wear was found in pHEMA (N_2_) (0.0197 mm^2^), even though its modulus was lower than that of pHEMA (air). This result may be explained by the bumpy surface of the air–polymerized hydrogel, which likely caused higher initial (run-in) wear. However, it is expected that once the protrusions are worn away, the wear rate of the (air) hydrogel will decrease. It should also be noted that the wear measurement technique had limitations. Significant drying of the PVA samples under ambient conditions made it difficult to compare the actual shape differences between the unworn and worn samples. This issue was not observed in the pHEMA hydrogels due to their lower water content. Therefore, direct wear comparison between PVA and pHEMA should be interpreted cautiously.

From the perspective of biochemical material characterization, in both PVA LM and PVA CP06 hydrogels, the swelling behavior adheres to first-order kinetics, with average *k*_1_ values of 0.16 ± 0.01 and 0.26 ± 0.1 h^–1^, respectively. Both hydrogels demonstrated substantial media absorption capacity within the initial 24 h,^44^ achieving 218.1 ± 4.1% for the PVA LM and 234.5 ± 7.0% for the PVA CP06, which is comparable to the results reported for freeze–thawed PVA hydrogels in PBS by Asy-Syifa et al.^64^ The pronounced swelling capacity of the PVA hydrogels is attributed to their hydrophilic nature and the porous three-dimensional (3D) structure formed through the freeze–thaw cycles during hydrogel preparation.^65^ Subsequently, the swelling ratio did not increase, indicating an equilibrium state.

Upon immersion in the aqueous medium, the dried poly­(vinyl alcohol) (PVA) chains are densely arranged. The SMSF infiltrates the polymer and initiates the formation of hydrogen bonds with the hydroxyl groups of PVA in the region with a low swelling ratio. As reported by Krzeminski et al.,^66^ the aqueous medium forms hydrogen bonds either with the hydroxyl groups located on the surfaces of polymer crystallites or with other available hydroxyl groups.^67^ Water that does not form hydrogen bonds with the PVA hydroxyl groups results in the formation of condensation water species, which are responsible for the increased values of equilibrium swelling and the formation of additional intra- and intermolecular hydrogen bonds. Furthermore, PVA hydrogels generally demonstrate enhanced water absorption in the presence of hyaluronic acid and improved lubrication properties, thereby mimicking the behavior of a natural joint.^35^ The LM and CP06 hydrogels vary in the methodology in preparation—the PVA CP06 hydrogels contain a single layer formed by two FT cycles. In contrast, the PVA LM hydrogels are formed by two layers, with the bottom layer formed by four FT cycles and the upper layer formed by CD. The maximum swelling ratio of the CP06 and LM hydrogels achieved after 72 h was 232.1 ± 6.8 and 215.6 ± 5.8%, respectively. This difference can be attributed to differences in the network structure and fabrication methods. It is well-established that the number of freeze–thaw cycles enhances crystallinity and cross-link density in the PVA hydrogels, forming a denser network that restricts water absorption.^68^ In the PVA LM, the base layer underwent four freeze–thaw cycles, resulting in reduced swelling compared to the PVA CP06. Additionally, the presence of an interfacial region between layers^69^ can hinder media diffusion and impose mechanical constraints, further limiting swelling.^44,70^ Previous studies have shown that prolonged freeze–thaw cycling modifies pore architecture,^71^ often resulting in fewer but larger pores and increased “freezable” water content, which collectively reduces overall swelling potential.^72^ Furthermore, hydrogels with higher cross-link density exhibit increased mechanical stiffness, resisting volumetric expansion under physiological-like osmotic conditions such as those in an SMSF.^73^

The low wear characteristics and structural stability observed in pHEMA hydrogels suggest that, despite their higher friction, these materials may serve as a robust platform for further material optimization. Future research may focus on enhancing their lubrication performance through the incorporation of biofunctional components such as hyaluronic acid or phospholipids, aiming to mimic the biochemical complexity of natural synovial environments. Such modifications could improve boundary lubrication and interfacial hydration without compromising the mechanical integrity of the base hydrogel. Given its resistance to long-term degradation under load, pHEMA may thus be particularly well suited for advanced tailoring toward cartilage replacement, where both mechanical resilience and adaptive lubrication are required.

Last but not least, some limitations of the study and future perspectives should be addressed. The use of a pin-on-plate configuration represents a simplified yet well-controlled tribological model that enables direct comparison of hydrogel systems under identical loading and lubrication conditions. However, it must be acknowledged that this setup does not reproduce the fully conforming geometry and interstitial fluid pressurization characteristic of native articular joints. In conforming contacts (e.g., ball-on-socket or condyle-on-plate), the load distribution and fluid film thickness differ markedly, which can influence both frictional response and wear mechanisms.^24,74–78^ Similar simplified configurations have been employed in several cartilage–hydrogel studies investigating boundary lubrication and wear mechanisms.^33–37^ These reports confirm that, despite the nonconforming geometry, physiologically relevant friction levels and reproducible trends can be achieved when model synovial lubricants and hydrated contact conditions are maintained. The present work should therefore be interpreted as a mechanism-oriented, comparative evaluation designed to identify structure–property relationships rather than to replicate the complex joint biomechanics. Future experiments from our group will extend toward conforming, curved geometries to verify the observed lubrication mechanisms under more physiological contact conditions.

Although cross-sectional wear area was assessed using optical scanning microscopy, the methodology was partially limited by surface drying and geometrical deviations, particularly in the convex PVA samples. These artifacts may have influenced the absolute surface area loss values and warrant careful interpretation. Future studies could benefit from the use of hydrated-state or in situ imaging techniques to obtain more robust wear measurements.

Furthermore, while fluid permeability is a known factor influencing interfacial lubrication and load support in hydrated biomaterials, no direct permeability measurements were performed in this study. Instead, permeability-related behavior was inferred from swelling data and literature-reported trends. Dedicated permeability characterization of the developed hydrogel systems, including pressure-driven or osmotic flow analysis, remains an important direction for future work to fully elucidate their lubrication mechanisms.

It should also be noted that this study focused on a simplified model synovial fluid. The use of more physiologically complete lubricants, including variable protein concentrations, hyaluronic acid, and phospholipid mixtures, may provide a more comprehensive understanding of hydrogel behavior in joint-mimicking environments. Future work could therefore explore the tribological response under progressively more complex biochemical conditions. In addition, extending the current setup toward higher contact pressures within the physiological range would allow replication of more realistic in vivo joint stresses and enable evaluation of hydrogel performance under demanding biomechanical loading scenarios.

## 5 Conclusion

This study provided a comparative analysis of PVA and pHEMA hydrogels under controlled cartilage-mimicking tribological conditions, focusing on how their chemistry, structure, and hydration influence mechanical and biotribological performance. The results represent relative material trends rather than absolute physiological predictions; however, they collectively form a consistent framework for understanding lubrication mechanisms in soft hydrogel systems. Key conclusions can be summarized as follows:

- PVA hydrogels demonstrated very low apparent friction (COF ≈ 0.006–0.01) under mild contact pressures (≈0.17 MPa), and high swelling ratios (≈230%) reflecting the contribution of their hydroxyl-rich, highly hydrated polymer network that supports boundary and hydration lubrication.
- Despite low-frictional behavior, PVA showed limited surface stability during extended articulation, indicating the need for mechanical reinforcement or composite design to improve long-term durability.
- pHEMA hydrogels exhibited higher friction coefficients (COF ≈ 0.02–0.09) but superior structural integrity and markedly lower wear, suggesting that their denser network, lower surface permeability, and higher cross-linking degree enhance resistance to plastic deformation and scratches.
- Quantitative roughness analysis confirmed that surface smoothness alone does not dictate frictional behavior. The tribological response is primarily governed by hydration capacity and chemical composition, not by topography.
- The overall results emphasize the trade-off between lubrication and mechanical stability, a key factor in designing cartilage-mimicking materials.

These results underscore the importance of balancing friction/lubrication and structural integrity in hydrogel design, and establish pHEMA as a mechanically resilient and chemically versatile platform for the development of advanced cartilage-mimicking systems, with high potential for further optimization through the incorporation of bioactive additives such as hyaluronic acid or phospholipids.

## Supplementary Material



## Data Availability

Supporting data associated with this article can be found online as Data set at Zenodo: DOI: 10.5281/zenodo.15053243.

## Notes

Correlation analyses linking swelling behavior, viscoelastic properties, and tribological performance across all hydrogel systems; extended descriptions of the DMA time-sweep analysis, friction and wear evaluation, and the complete set of correlation plots presented (Figure S1) (PDF)
