# Supplementary material for: Toward Cartilage-Mimicking Biomaterials: Biotribological, Biochemical and Structural Evaluation of pHEMA and PVA-Based Hydrogels
Source: ACS Omega. 2025 Dec 8;10(51):63441–54. doi: 10.1021/acsomega.5c10283 (PMC12756760; doi:10.1021/acsomega.5c10283)
Supplement: Supplementary file 1 [file ao5c10283_si_001.pdf]

Towards cartilage-mimicking biomaterials:

Biotribological, biochemical and structural evaluation of pHEMA and PVA-based hydrogels

David Nečas[1,*], Daniel Něměček[1], Jan Gregora[1], David Rebenda[1], Zuzana Kadlecová[2], Ivana Chamradová[2], Monika Trudičová[2,3], Pavel Čípek[1], Petr Čípek[1], Ladislav Šnajdárek[1], Lucy Vojtová[2], Martin Vrbka[1], Ivan Křupka[1], Martin Hartl[1]

[1] Department of Tribology, Faculty of Mechanical Engineering, Brno University of Technology, Technická 2896/2, 616 69 Brno, Czech Republic

[2] Advanced Biomaterials, Central European Institute of Technology, Brno University of Technology, Purkyňova 656/123, 612 00 Brno, Czech Republic

[3] Materials Research Center, Faculty of Chemistry, Brno University of Technology, Purkyňova 464/118, 612 00 Brno, Czech Republic

[*]Corresponding author, Tel.: +420 541 143 239, E-mail address: David.Necas@vut.cz

Supporting Information

S1. Correlation between swelling, stiffness, friction and wear

To better understand the relationship between intrinsic material parameters and frictional response, correlation plots were constructed using the data obtained from swelling experiments (Section 3.1), DMA time-sweep analysis (Section 3.1), and tribological testing (Section 3.2). *Fig. S1* (top) shows how the coefficient of friction (COF) relates to equilibrium swelling capacity S_{max} and complex modulus E* across all hydrogel systems. COF values were extracted as an average from the mid-region of the reciprocating test (cycles 200–1,800 for cartilage and cycles 1,000–18,000 for CoCrMo pin), where stable friction conditions were observed. Complex modulus values represent the average E* obtained from DMA time-sweep measurements over the entire time range. These correlations suggest that a general trend of lower friction with increasing swelling and moderate stiffness can be observed, especially for PVA-based hydrogels. However, this relationship is not universal, as pHEMA air also exhibited relatively low COF despite its limited swelling capacity. This highlights the importance of material-specific surface properties and lubrication mechanisms beyond bulk hydration alone.

In addition to frictional behavior, wear was plotted against both equilibrium swelling and complex modulus to evaluate potential structure–wear relationships. As shown in *Fig. S1* (bottom), PVA-based hydrogels with high swelling ratios also exhibited higher wear, suggesting a potential trade-off between interfacial lubrication and bulk durability. Conversely, pHEMA hydrogels, despite their relatively low swelling, maintained substantially lower wear volumes, particularly for the N_2-polymerized variant. The plot of wear versus complex modulus E* reveals a trend of increasing wear with increasing stiffness across the tested hydrogel systems. This observation suggests that higher stiffness may compromise the material's ability to conform to the counterface, leading to greater abrasive interactions under load. While such a trend contrasts with conventional expectations from chemically crosslinked networks, it highlights the complex interplay between mechanical integrity, surface compliance, and wear behavior. Moreover, outliers in the dataset indicate that additional

factors such as surface microstructure, crosslinking heterogeneity, and lubrication regime likely contribute to the overall wear resistance.

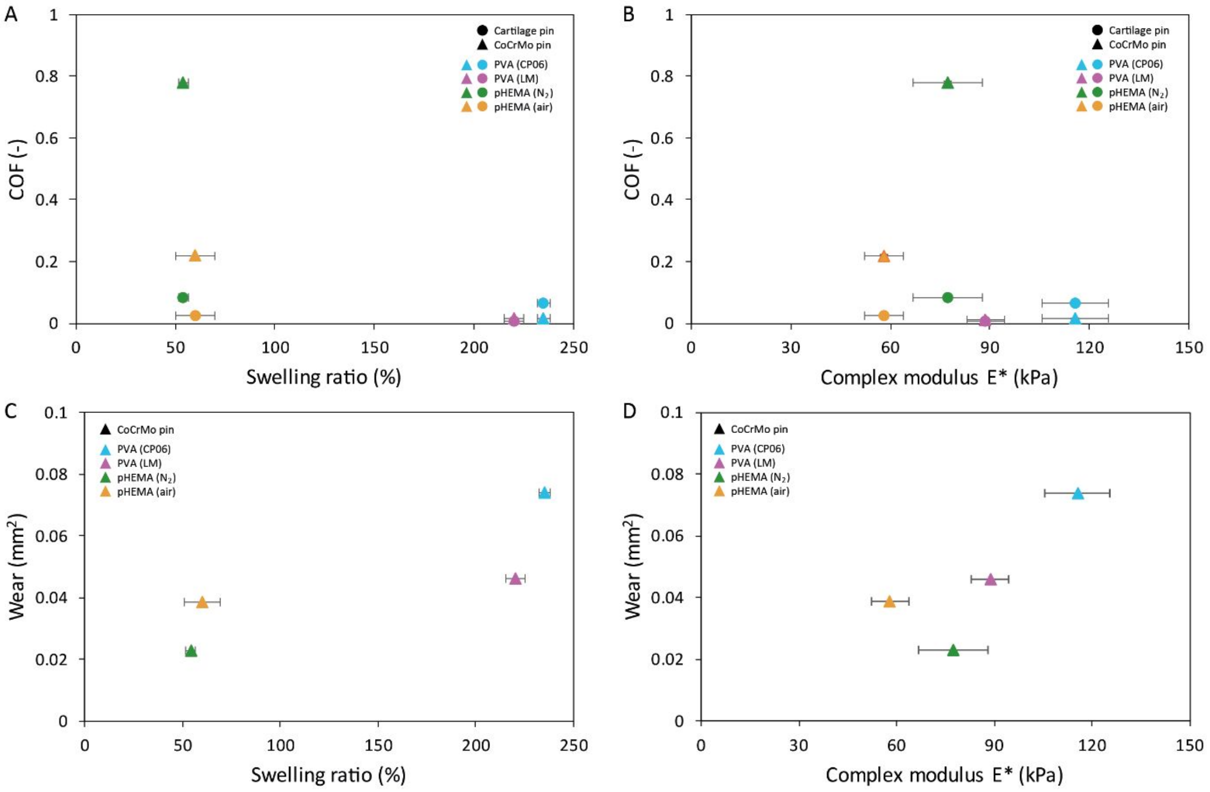

Figure S1. Top: Correlation between equilibrium swelling ratio S_{max} (A), complex modulus E* (B), and coefficient of friction (COF). Bottom: Correlation between equilibrium swelling ratio S_{max} (C), complex modulus E* (D), and wear. *Vertical error bars (standard deviations) are not displayed as they fall within the symbol size and are indistinguishable at the current graph scale.**
